# Blockade of Hemichannels Normalizes the Differentiation Fate of Myoblasts and Features of Skeletal Muscles from Dysferlin-Deficient Mice

**DOI:** 10.3390/ijms21176025

**Published:** 2020-08-21

**Authors:** Luis A. Cea, Gabriela Fernández, Guisselle Arias-Bravo, Mario Castillo-Ruiz, Rosalba Escamilla, María C. Brañes, Juan C. Sáez

**Affiliations:** 1 Instituto de Ciencias Biomédicas, Facultad de Ciencias de la Salud, Universidad Autónoma de Chile, Santiago 8910060, Chile; gabrielafernandezcampos@gmail.com (G.F.); guisselle.arias.b@gmail.com (G.A.-B.); 2 Escuela de Química y Farmacia, Facultad de Medicina, Universidad Andres Bello, Santiago 8370149, Chile; mhcastilr@gmail.com; 3 Departamento de Ciencias Químicas y Biológicas, Facultad de Ciencias de la Salud, Universidad Bernardo O Higgins, Santiago 8370854, Chile; 4 Instituto de Neurociencias, Centro Interdisciplinario de Neurociencias de Valparaíso, Universidad de Valparaíso, Valparaíso 2340000, Chile; rescamhdz@yahoo.com; 5 Consorcio de Investigación Naturalis SA, Santiago 8700000, Chile; mcbranes@gmail.com; 6 Departamento de Fisiología, Facultad de Ciencias Biológicas, Pontificia Universidad Católica de Chile, Santiago 8320000, Chile

**Keywords:** PPARγ, lipid accumulation, connexons, sarcolemma permeability, hemichannel blocker, creatine kinase, muscular dystrophy

## Abstract

Dysferlinopathies are muscle dystrophies caused by mutations in the gene encoding dysferlin, a relevant protein for membrane repair and trafficking. These diseases are untreatable, possibly due to the poor knowledge of relevant molecular targets. Previously, we have shown that human myofibers from patient biopsies as well as myotubes derived from immortalized human myoblasts carrying a mutated form of dysferlin express connexin proteins, but their relevance in myoblasts fate and function remained unknown. In the present work, we found that numerous myoblasts bearing a mutated dysferlin when induced to acquire myogenic commitment express PPARγ, revealing adipogenic instead of myogenic commitment. These cell cultures presented many mononucleated cells with fat accumulation and within 48 h of differentiation formed fewer multinucleated cells. In contrast, dysferlin deficient myoblasts treated with boldine, a connexin hemichannels blocker, neither expressed PPARγ, nor accumulated fat and formed similar amount of multinucleated cells as wild type precursor cells. We recently demonstrated that myofibers of skeletal muscles from blAJ mice (an animal model of dysferlinopathies) express three connexins (Cx39, Cx43, and Cx45) that form functional hemichannels (HCs) in the sarcolemma. In symptomatic blAJ mice, we now show that eight-week treatment with a daily dose of boldine showed a progressive recovery of motor activity reaching normality. At the end of this treatment, skeletal muscles were comparable to those of wild type mice and presented normal CK activity in serum. Myofibers of boldine-treated blAJ mice also showed strong dysferlin-like immunoreactivity. These findings reveal that muscle dysfunction results from a pathophysiologic mechanism triggered by mutated dysferlin and downstream connexin hemichannels expressed de novo lead to a drastic reduction of myogenesis and favor muscle damage. Thus, boldine could represent a therapeutic opportunity to treat dysfernilopathies.

## 1. Introduction

Muscular dystrophies induced by mutations in *DYSF* gene, which encodes a protein named dysferlin, leads to the complete absence of the protein and are grouped as dysferlinopathies. These diseases present a late-onset (around the second and third decade of life) and patients manifest progressive deterioration of skeletal muscles first affecting limb-girdle muscles but then compromising the majority of skeletal muscles. Currently, dysferlinopathies are untreatable possibly due to the limited knowledge of relevant molecular targets.

It is accepted that dysferlin participates in membrane repair processes after damage, which are activated by an increase of the intracellular amount of cytoplasmic Ca^2+^ at the damaged zone [1]. However, the recovery of membrane repairing function is achieved with myoferlin, another protein that plays a similar role as dysferlin, or by expressing the domain of dysferlin in charge of membrane repair (mini-dysferlin), but does not arrest the muscular degeneration [2]. Thus, the involvement of an additional and critical pathological mechanism in muscular degeneration remains unknown. A peculiar change of dysferlin deficient skeletal muscles that remains without explanation is the accumulation of fat tissue [3,4]. In this regard, we have suggested that de novo expression of connexin hemichannels (Cx HCs) might participate in muscle changes of dysferlinopathies as a plausible hypothesis [5].

The involvement of Cx HCs in skeletal muscle deterioration induced by several noxious conditions (e.g., denervation, bacterial lipopolysaccharide-induced endotoxemia, and long-term glucocorticoids treatment) has been demonstrated [6,7,8]. Under these conditions, Cx HCs permeable to Ca^2+^ facilitate the increase in basal intracellular free-Ca^2+^ concentration in myofiber, leading to activation of Ca^2+^-dependent proteases promoting atrophy. In these studies, the relevance of Cx43 and Cx45 HCs was demonstrated using animals deficient in the expression of these two proteins in differentiated myofibers (Cx43^fl/fl^Cx45^fl/fl^:Myo-Cre mice). Alternatively, the high Cx HC activity could be reduced with classic gap junction channel inhibitors such as carbenoxolone and 18β-glycyrrhetinic acid [9]. However, gap junction channels are essential for the normal functioning of several vital tissues (e.g., heart) and consequently, the long-term use of them in chronic diseases, such as those caused by gene mutations, could be deleterious. Alternatively, compounds that block Cx HCs but do not affect gap junction channels could be effectively used. An example of those compounds is boldine, an alkaloid that can be extracted from *Peumus boldus* tree called Boldo, which has been shown to block Cx HCs but does not inhibit gap junction channels formed by Cxs [7,10,11]. Accordingly, long-term treatment of chronic diseases such as Alzheimer’s Disease and diabetes does not induce undesired side-effects [10,11].

To study the possible role of Cx HCs in dysferlinopathy, we used boldine as a potential therapeutic drug, in dysferlin deficient myoblasts, and in blAJ mice, the animal model of dysferlinopathies [12]. Dysferlin deficient myoblasts induced to differentiate into myogenic lineage were found to express PPARγ, contained fat, and within 48 of differentiation formed fewer multinucleated cells. Moreover, skeletal myofibers from symptomatic blAJ mice expressed Cx HCs that increased sarcolemmal permeability, elevated the basal intracellular Ca^2+^ signal of myofibers and promoted several other changes characteristic of dysferlinopathies including histological, biochemical, and functional alterations. However, all changes described above were reverted to normality in symptomatic blAJ mice treated for several weeks with boldine. Interestingly, the dysferlin-like reactivity was detected in myofibers of boldine-treated bIAJ mice, suggesting Cx HCs as part of a novel pathophysiologic mechanism that explains many features of dysferlinopathies. Besides, our results provide a proof of concept for the use of Cx HC inhibitors as therapeutic agents to alleviate muscle dystrophy caused by mutated dysferlin.

## 2. Results

### 2.1. Boldine Rectifies the Aberrant Adipogenic Commitment of Dysferlin-Deficient Myoblasts

**Figure 1:**
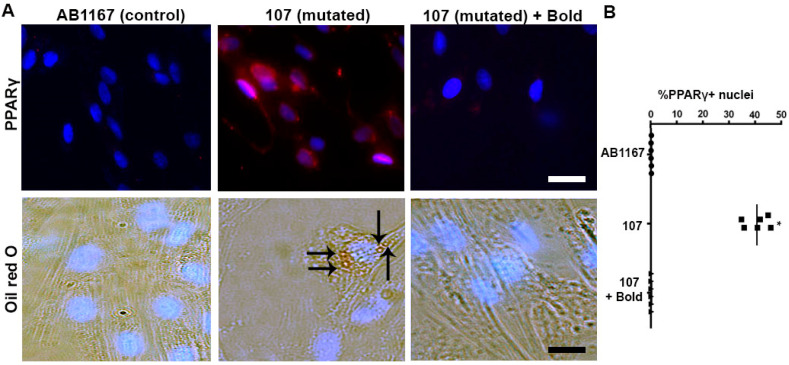
Boldine rectifies the adipogenic commitment fate of dysferlin-deficient myoblasts. Human myoblasts cell lines were induced to differentiate into myotubes. (**A**) Top panels, immunofluorescence analysis of PPARγ (red signal) and nuclei (DAPI, blue signal) in AB1167 (control) and 107 (dysferlin mutated) cells (treated or not with boldine) cultured for 10 h in myogenic differentiation media. Bottom panels, AB1167 and 107 cells were cultured for 6 days in myogenic differentiation media and were analyzed with oil red O staining (arrows) for triglycerides accumulation. In parallel, 107 cells were treated or not with 50 μM boldine for 6 days. Scale Bar: 10 μm. (**B**) Quantification of PPARγ positive nuclei expressed as percentage (% PPARγ + nuclei) from 10 fields like in (**A**). Each value represents the mean ± SEM, *n* = 6 cell cultures, * *p* < 0.05.

A characteristic histological feature of dysferlinopathies is the accumulation of fat in skeletal muscles [13,14] and eventually the replacement of muscle fibers by adipose cells [4]. Since we have previously found high activity of Cx HCs in a dysferlin deficient human cell line [5] and dysferlin deficient patients and skeletal muscles of blAJ mice present fat accumulation [13,14,15], we speculated that some muscle precursor cells might acquire adipogenic commitment due to the elevated Cx HC activity. To test this possibility, we used human myoblast cell lines derived from normal (AB1167) and dysferlin mutated (107 cells) patients. While these cells were induced to acquire myogenic commitment, sister cultures were treated with 50 μM boldine, a blocker of Cx43 HCs [10,11] and Cx45 HCs but not Cx39 HCs (Supplementary Figure S1). After 10 h in differentiation medium, many 107 cells were PPARγ positive (Figure 1A, top middle panel) and ~40% presented nuclear PPARγ reactivity (Figure 1B), which is expressed by cells committed for adipogenesis [16]. In contrast, 107 cells treated with boldine showed undetectable PPARγ reactivity similar to images seen in control cells (Figure 1A, top right panel and top left, respectively).

Besides, and consistent with the finding that myoblasts acquired the adipogenic commitment, several 107 cells contained triglyceride accumulation as denoted by their oil red O positive staining at day 6 of differentiation (Figure 1A, bottom middle panel). Whereas, AB1167 cells or 107 cells treated with boldine remained negative for oil red O staining (Figure 1A, bottom left and right panels, respectively).

### 2.2. Connexin Hemichannels Are Involved in the Accumulation of Fat within the Skeletal Muscles of blAJ Mice

**Figure 2:**
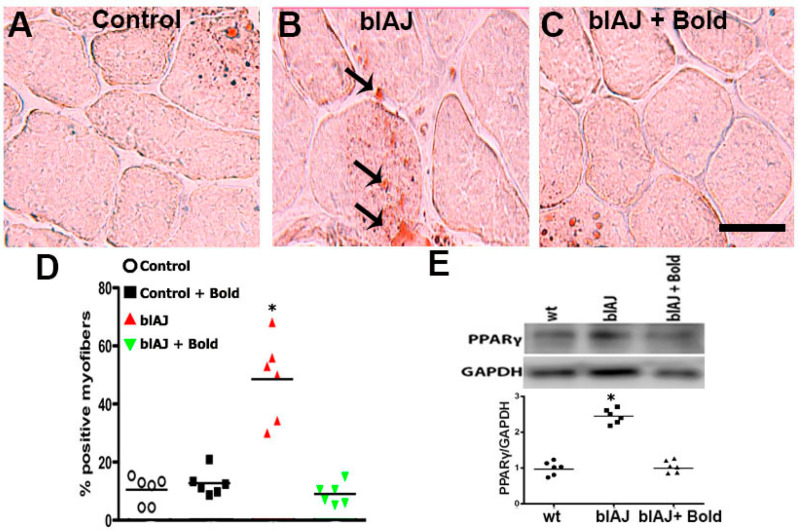
Boldine abrogates the fat accumulation in skeletal muscles from dysferlin deficient mice. GC muscles were dissected from (**A**) control, (**B**) dysferlin-deficient mice (blAJ) and (**C**) blAJ mice treated with boldine (100 mg/kg daily during 8 weeks) and the fat accumulation was evaluated by oil red O staining (arrows). (**D**) Graph showing the quantification (%) of oil red O positive cells from field images as shown in A, B, and C. Each value of the graph represents the mean ± SEM. *N* = 6 animals per condition. (**E**) Top panel, Western blot analysis of the relative amount of PPARγ in GC muscles from wt, dysferlin deficient (blAJ), and blAJ mice treated with boldine (daily, 100 mg/kg) by 8 weeks. Bottom panel, graph showing the densitometric analysis of PPARγ band normalized by the intensity of the GAPDH band. *N* = 6 animals for each condition. * *p* < 0.05 respect to all studied conditions. Scale bar = 50 μm.

Since we have recently shown that Cx43/Cx45 expression deficient blAJ myofibers do not present lipid accumulation in skeletal muscles [15] and boldine treatment normalized the fate of differentiation of human myoblasts (see above), we decided to evaluate whether boldine could abrogate the fat accumulation in muscles of blAJ mice. To this end, 8-week old blAJ mice were treated daily for 8 weeks with boldine (100 mg/kg daily). Then, cross-sections of Gastrocnemius (GC) muscles were analyzed using oil red O staining. Representative images in Figure 2A–C show that evident positive staining was only detected in muscle sections from blAJ mice under no treatment (Figure 2B) but those from blAJ mice treated with boldine (Figure 2C) or from normal mice (Figure 2A), were fairly negative. These qualitative observations were supported by quantification of the number of positive cells in each condition (Figure 2D). Consistent with the presence of fat in muscles of blAJ mice, we also found a high relative amount of PPARγ in these muscles (Figure 2E). More importantly, the amount of PPARγ detected in GC muscles from blAJ mice treated for 8 weeks with boldine was as low as in muscles from wild type mice treated or not with boldine (Figure 2E).

### 2.3. Boldine Favors Dysferlin-Deficient Myoblasts Differentiation to Myotubes

**Figure 3:**
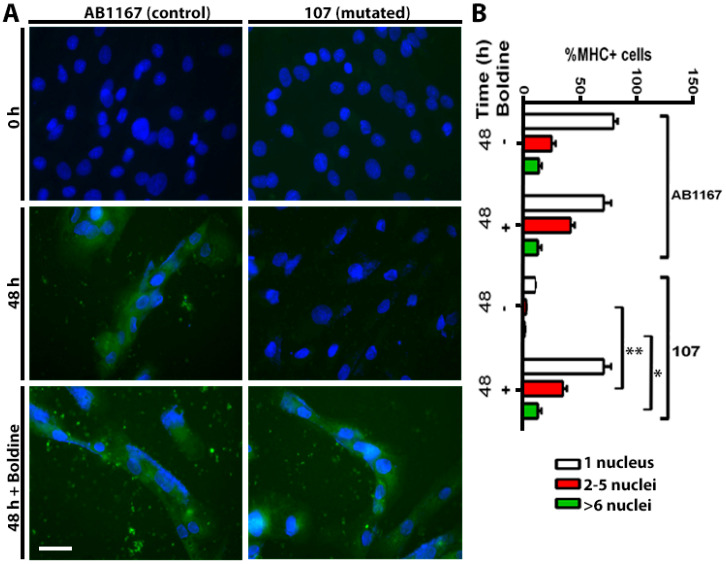
Boldine normalizes the delayed multinucleated cell formation in human myoblasts expressing mutated dysferlin. Human myoblast cell line cultures were induced to differentiate in multinucleated cells. (**A**) AB1167 (control) and 107 (dysferlin mutated) cells were cultured for 48 h in myogenic differentiation media in the absence or presence of 50 μM boldine. Multinucleated cells were recognized by their MHC immunoreactivity. Scale bar: 20 µm (**B**). The percentage of MHC positive cells is presented (%MHC+ cells) and the number of nuclei contained per positive MHC cell was categorized as cells presented 1, 2–5 or more than 6 nuclei. *N* = 6 cell cultures. * *p* < 0.05, 107 cells with 2–5 nuclei versus 107 cells plus boldine with 2–5 nuclei. *N* = 6 cell cultures. ** *p* < 0.01, 107 cells with > 6 nuclei compared with 107 cells plus boldine with > 6 nuclei. There is no significant difference between 48 h cultured 107 cells plus boldine and AB1167 (control) cells.

Since skeletal muscles of blAJ mice with myofibers deficient in Cx43/Cx45 expression present a low number of myofibers with internal nuclei and small CSA and low activity of serum CK [15], we thought that inhibition of Cx HCs with boldine might render a similar outcome as silencing Cx43 and Cx45 expression. To test this possibility, we first used AB1167 and 107 cells, which were induced to differentiate in the absence or presence of boldine (30 μM). Forty-eight hours later, multinucleated cells were recognized by their MHC immunoreactivity and the number of nuclei per cell was quantified (Figure 3). AB1167 cells cultured with or without boldine presented a similar number of multinucleated cells, whereas cultures of 107 cells presented very few multinucleated cells (Figure 3). Notably, in cultures of 107 cells induced to differentiate in the presence of boldine, the number of multinucleated myotubes was similar as that found in cultures of AB1167 cells (Figure 3A, bottom right). These findings suggest that Cx43 and Cx45 HCs drastically reduce the full differentiation of myotubes that is characterized by the fusion of myotubes and multinucleated cells formation [16].

### 2.4. Blockade of Functional Connexin Hemichannels Restores the Sarcolemma Permeability Features of Skeletal Muscles from blAJ Mice

Since human myoblasts bearing dysferlin mutations express functional Cx HCs [5], and the Cx43 and Cx45 silencing in blAJ myofibers prevents muscle degeneration [15], we wondered if blockade of Cx43 and Cx45 HCs could improve different abnormalities of skeletal muscles from symptomatic blAJ mice. In favor of this possibility, we first found that the Cx HC activity of myofibers from blAJ mice was drastically reduced to normal values both in vivo and in vitro by boldine treatment (Supplementary Figures S2 and S3A). Concordantly with the positive or negative EB^−4^ staining of myofibers from blAJ or control mice, respectively [15] (Supplementary Figures S2 and S3A). In the present work, the intracellular EB^−4^ staining was undetectable or absent in myofibers from blAJ mice treated with boldine for eight weeks as it was observed in myofibers from controls or controls treated with boldine. (Supplementary Figure S2). On the contrary, EB^−4^ staining was evident in the cytoplasm of blAJ myofibers (Supplementary Figure S2). We also found that the Etd^+^ uptake of isolated blAJ myofibers was completely blocked by the acute application of 30 µM boldine or 50 µM carbenoxolone (Cbx) (Supplementary Figure S3A,B, respectively), a hemichannel blocker [9]. However, no additional effect of Cbx over that induced by boldine was detected (Supplementary Figure S3B).

In addition, after the above verification of an effective Cx HC blockade with boldine in myofibers from blAJ mice, the previously reported increase in cytoplasmic Ca^2+^ signal of myofibers from blAJ mice [15] was not detected in control myofibers nor in myofibers from blAJ mice treated with boldine (Supplementary Figure S3C). Thus, this finding strongly suggests that abolishing Cx HCs functional state is effective in reverting this feature of dysferlinopathies.

### 2.5. Boldine Induces Progressive Recovery of Motor Performance and Abrogates Muscle Damage in Symptomatic blAJ Mice

**Figure 4:**
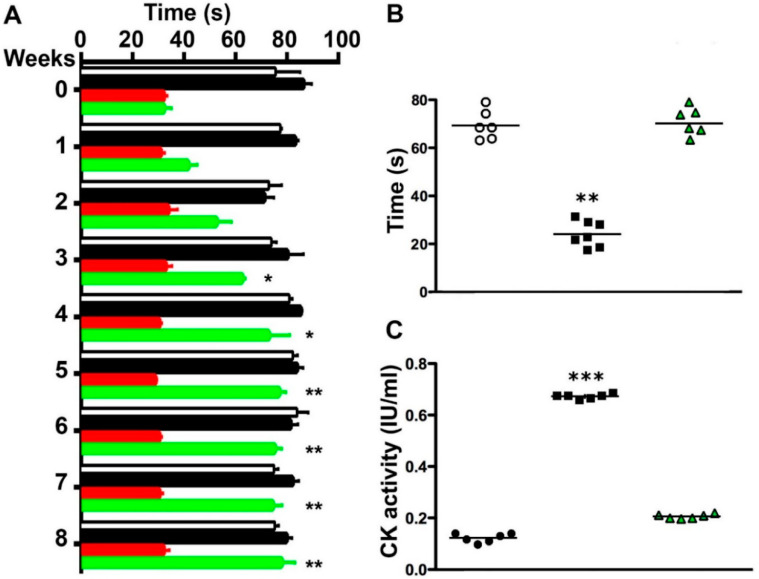
Boldine normalizes the muscle performance and serum creatine kinase activity of symptomatic dysferlin deficient mice. To evaluate motor performance, four groups of 8 month-old mice were used. (1) Control mice (white bars), (2) control mice treated with boldine (black bars), (3) blAJ mice (red bars), and (4) blAJ mice treated with boldine (green bars). The muscle performance of all mice was evaluated over the following 8 weeks using two motor training tests, (**A**) the rotator test and (**B**) the four limbs hanging test (* *p* < 0.05 and ** *p* < 0.01). Each value represents the mean ± SEM. N = 6. (**C**) Creatine kinase activity (CK, expressed as international units, IU per milliliter) was evaluated in serum obtained from blAJ mice, blAJ mice treated for 8 weeks with boldine (blAJ + boldine) and control mice (Control). Each plotted value corresponds to the mean ± SEM. *N* = 6 animals for each condition, in (**A**). * *p* < 0.05 and ** *p* < 0.01 blAJ + Bold respect to blAJ, no significant difference between blAJ + Bold and Control or Control + Bold. In (**B**), ** *p* < 0.01 respect to all conditions. In (**C**), *** *p* < 0.001 respect to all conditions.

Since several abnormalities present in dysferlinopathies are mediated by Cx HCs and bIAJ mice with skeletal myofibers deficient in Cx43/Cx45 expression do not manifest deficient motor performance [15], it was plausible that inhibition of these channels could improve functional recovery of mice with dysferlinopathy. This possibility was evaluated using two motor performance tests, the rotarod test and the hanging test, applied to symptomatic 8-month-old blAJ mice. We found that blAJ mice presented ~70% less performance than control mice (measured as the duration (in seconds) that animals remained on rotarod) (Figure 4A). As expected, blAJ mice treated with boldine showed progressive improvement of performance, reaching complete recovery after 4 weeks of treatment that persisted throughout the experiment (8 weeks) (Figure 4A). In the hanging test assessed to 8-month-old blAJ mice treated with boldine for 8 weeks, animals resisted ~30% more of the time hanging from the horizontal wire as compared to control mice, whereas blAJ mice treated with boldine remained hanged for as long as control mice did (Figure 4B). Boldine did not affect the performance of control mice (Figure 4A), suggesting it could be a safe new pharmacological therapy to treat dysferlinopathies.

We have shown that mouse myofibers bearing mutated dysferlin present an elevated basal Ca^2+^ signal due to high Cx HCs activity in the sarcolemma [5,15] (Supplementary Figure S3). In addition, a persistent elevated intracellular free Ca^2+^ concentration could lead to the death of skeletal myofibers [17] and in dysfernilopathy this is reflected by elevated serum creatine kinase activity (CK) [18,19]. Therefore, we decided to analyze whether inhibition of Cx HCs with boldine affects the serum CK activity in symptomatic blAJ mice. In serum samples, we found significantly higher CK activity in blAJ mice as compared to control mice (Figure 4C) but in bIAJ mice treated with boldine during the last 8 weeks, the CK activity was similar to that of control mice (Figure 4C). As mentioned above, the increase in CK reflects myofiber death, which promotes muscle regeneration evidenced by the presence of myofibers with smaller cross-sectional area (CSA) and increased number of myofibers with internal nuclei as it has been documented [20]. Thus, we studied whether these parameters can be normalized in GC muscles by treating 8-month-old blAJ mice with boldine. We found a significant increase in the number of myofibers with more than one internal nucleus (Supplementary Figure S4A,B) and a significant increase in myofibers with smaller CSA (Supplementary Figure S4C) as compared to muscles from control mice. However, and consistent with the normal motor performance, in muscles from blAJ mice daily treated during 8 weeks with boldine, the number of myofibers with internal nuclei and smaller CSA were comparable to those of muscles from control mice of the same age not treated or treated with boldine (Supplementary Figure S4C).

### 2.6. Dysferlin-Like Immunoreactivity Is Recovered in Skeletal Muscles of blAJ Mice Treated with Boldine

**Figure 5:**
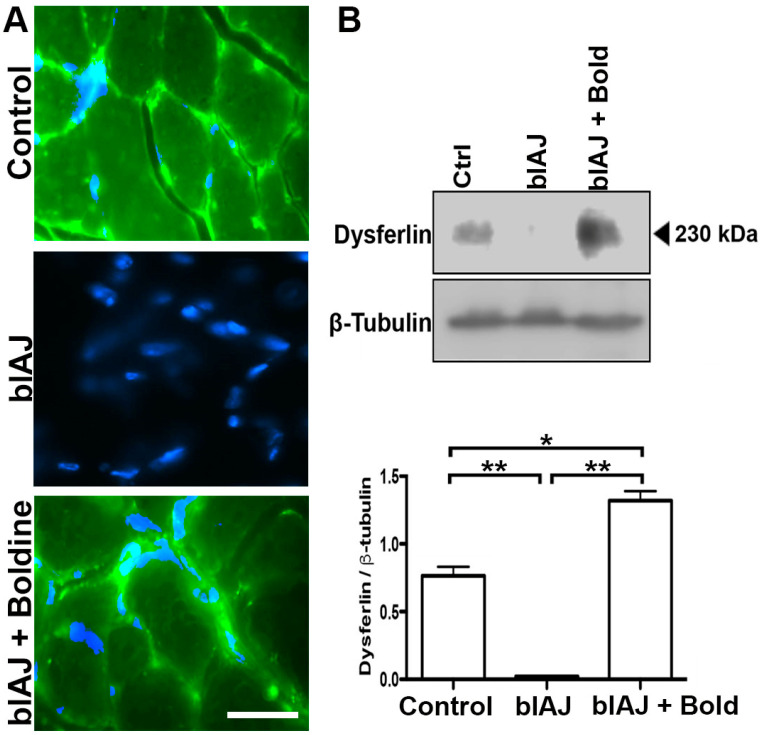
Treatment with boldine induces the recovery of dysferlin immunoreactivity in muscles of blAJ mice. (**A**) The presence, cell distribution, and relative amount of dysferlin were evaluated by immunofluorescence analysis in GC muscles cross-sections from control, blAJ and blAJ treated with boldine mice. Nuclei were stained with DAPI (blue signal). Scale bar = 50 μm. (**B**) The relative amount of dysferlin was evaluated in the above muscles by Western blot analysis, using Β-tubulin as a loading control. Densitometry analysis of the ~230 kD band was performed in 6 independent experiments and the mean ± SEM is presented in the graph. * *p* < 0.05 and ** *p* < 0.01. *n* = 6 animal for each condition.

We have previously detected a dysferlin-like immnunoreactivity in muscles of blAJ mice with myofibers deficient in Cx43 and Cx45 expression [15]. blAJ mice present a retrotransposon in intron 4 [21] and the dysferlin reactivity might derive from an aberrant transcript corresponding to exons 5–55, which in the absence of boldine is quickly degraded by Ca^2+^-dependent nucleases [22]. Since we found that inhibition of Cx HCs with boldine normalized the Ca^2+^ signal of blAJ myofibers, which could prevent the activation of Ca^2+^-dependent hydrolases [23], it was plausible that muscles of blAJ mice also show a dysferlin reactivity. By immunofluorescence and Western blot analysis, we detected a dysferlin-like reactivity in skeletal muscles of blAJ mice bearing a normalized cytoplasmic Ca^2+^ signal after boldine treatment. In skeletal muscle samples from blAJ mice, dysferlin was undetectable by both techniques (Figure 5A,B). In contrast, dysferlin-like reactivity was detected in the cell periphery in the cross-section of muscles from control mice (Figure 5A). Surprisingly, in muscles of boldine-treated blAJ mice, a dysferlin-like reactivity was also detected mainly in the cell periphery (Figure 5A). Notably, in the latter muscles, a high amount of total dysferlin-like band was detected at ~230 kDa that was more intense than the dysferlin band detected in control muscles (Figure 5B).

## 3. Discussion

**Figure 6:**
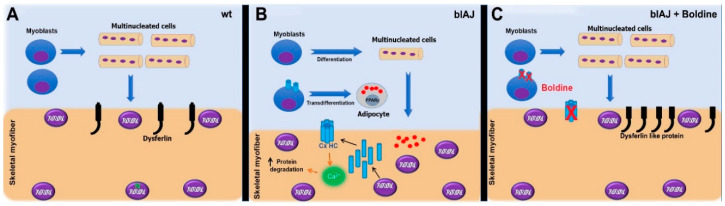
Proposed model. (**A**). Control myoblasts undergo differentiation and form multinucleated cells, which later on form skeletal myofibers with peripheral nuclei and dysferlin protein presented as a black protein in the membrane. (**B**). In blAJ mice, some myoblasts carrying a mutated dysferlin form fewer multinucleated cells as compared to controls whereas others undergo transdifferentiation to adipocytes leading to fat accumulation (red dots). The absence of dysferlin induces the expression of connexin hemichannels (Cx HC), which in turn enable an increase in intracellular Ca^2+^ signal, increasing the protein degradation promoting the muscle atrophy (skeletal myofiber of smaller diameter) and degradation of mutated dysferlin (absence). These skeletal myofibers also show many nuclei internally located. (**C**). In blAJ mice treated with boldine (blAJ + boldine), all these changes are reverted by boldine, a connexin hemichannel (Cx HC) inhibitor (X). In these myofibers, a dysferlin-like protein of unknown functional activity is detected.

In this work, we demonstrated that about 40% of human myoblasts bearing mutated dysferlin (107 cells) acquire adipogenic commitment and consequently form much fewer multinucleated cells upon induction to myogenic differentiation for 48 h. Notably, these alterations were corrected by boldine, both in 107 cells and in skeletal muscles of symptomatic blAJ mice. Consequently, boldine treatment normalized different alterations of blAJ myofibers mice completely recovering their motor function (Figure 6).

Fatty infiltration, a hallmark of dysferlinopathy, can be evidenced through magnetic resonance analysis [3] or by lipid staining in biopsies of skeletal muscles [13,14]. However, its genesis remained rather unknown. Consequently, with the human disease, eight-week old blAJ mice are symptomatic and present fat accumulation in their skeletal muscles [13]. We now found that 107 cells, a human dysferlinopathy myoblast cell line, cultured under conditions that promote the acquisition of myogenic commitment, present a relevant number of cells with aberrant adipogenic commitment (PPARγ expression), which explains the elevated number of oil red O positive cells and the reduced number of myotubes found at this late stage of differentiation. In the same line of analysis, we also found a high amount of PPARγ in muscles of adult blAJ mice (18-week old), indicating that an important number of myoblasts kept acquiring an adipogenic commitment. Also, this finding could explain the high fat (oil red O positive cells) content found in muscles of blAJ mice, some of which might correspond to adipocytes. Interestingly, boldine abrogated cells from acquiring the adipogenic commitment by reducing to normal values the amount of PPARγ and consequently reducing the fat generation both in vitro and in vivo.

A relevant question that remains to be answered is: Which cell alterations are induced by mutated dysferlin that could explain the elevated Cx HC activity of myoblasts? Since dysferlin plays a relevant role in membrane trafficking [24], a speculative explanation might be that a reduced retrieval of Cx HCs from the cell membrane increases the number of functional hemichannels. This could result in a modified exchange of ions, metabolites, and cell signals across the cell membrane, preventing the normal metabolic reprogramming of stem cells [25] and affecting the expression of master genes for cell differentiation. Concerning the latter, reactive oxygen species (ROS) are among the putative molecules that activate Cx HCs [26] and induce adipogenic transdifferentiation [27]. Since the gain of function of Cx HCs could lead to ROS generation [28], it is conceivable that the elevated Cx HC activity of myoblasts 107 could contribute to adipogenic transdifferentiation. In support of this mechanism of a switch in gene expression and commitment for differentiation, the deletion of MyoD using CRISPR/Cas-9 in a myoblast cell line (C_2_C_12_ cells) induces adipogenic transdifferentiation [29]. Consistent with the above possibility, the inhibition of Cx HCs with boldine favors the generation of myotubes in vitro and fully differentiated myofibers with peripheral nuclei and normal CSA in vivo. It remains to be studied whether the fat found in muscles of eight-week-old blAJ mice correspond to fully and normally differentiated adipose tissue and whether it vanished upon boldine treatment due to its use as energy fuel for muscle activity.

Interestingly, the attenuated response of muscle recovery after induction of regeneration by notexin-induced muscle damage in a mouse model of dysferlinopathy (C57BL/10.SJL-Dysf) [30] is in agreement with our findings in blAJ mice. However, in C57BL/10.SJL-Dysf mice, the general pattern of expression of various markers of satellite cell activation and differentiation were not significantly disturbed, suggesting that the defect in regeneration is not underlaid by a satellite cell defect. Moreover, C57BL/10.SJL-Dysf myoblasts of notexin injured muscles fused and formed myotubes effectively [30], and the difference in the regenerative process was suggested to be more related to a failure of clearance of necrotic tissue by inflammatory cells rather than the ability to form new desmin positive fibers [30]. This apparent controversy might be explained by differences between ontogeny and regeneration and or differences in the methodology used to evaluate the formation of myotubes. Further studies are required to clarify this issue.

Despite skeletal myofibers of blAJ mice expressing three Cxs (39, 43 and 45), the consequences described here seem to be mainly explained by blockade of only Cx43 and Cx45 HCs. In favor of this statement, we demonstrated that boldine treatment prevented completely the increase in sarcolemma permeability of myofibers to EB^4-^ in vivo and to Etd in vitro. The EB^4-^ uptake has been previously shown to reflect membrane permeabilization rather than membrane breakdown. Under several conditions, it has been observed that fluorescent dextran of about 10 kDa, which does not permeate Cx HCs, does not label myofibers that express Cx HCs [6,8]. In addition, boldine blocks Cx43 HCs [10,11] and Cx45 but not Cx39 HCs as shown herein using HeLa cells transfectants exposed to DCFS. Furthermore, the outcome of boldine treatment described here highly resembles the outcome of silencing just Cx43 and Cx45 in myofibers of blAJ mice [15]. Although dysferlin is also expressed in other tissues [31], the most relevant manifestation is muscular weakness. This is in agreement with the equivalent recovery observed after treatment with boldine that could block hemichannels in every affected organ presenting similar alterations (i.e., heart and brain) or silencing Cx43 and Cx45 expression only in myoblast of skeletal muscles.

Several conditions could contribute to maintaining Cx HCs open in myoblasts and myofibers. For instance, a moderate increase in intracellular Ca^2+^ signal enhances the activity of Cx43 HCs [32] and Cx43 and Cx45 HCs are activated by reduced redox potential and nitrosylation [33,34]. Moreover, an activated inflammasome, as it occurs in dysferlinopathy, generates pro-inflammatory cytokines [35], which also increase the activity of Cx HCs [7,36]. Considering that the gain of function of Cx HCs could lead to the generation of reactive oxygen species [28], it is conceivable that these reactive by products reduce the MyoD expression [27,37], a transcriptional factor that promotes the myogenic commitment of satellite cells [38], allowing the expression of adipogenic proteins like PPARγ, which control the adipogenic differentiation [39].

In addition to the above-mentioned possibilities, the expression of Cxs by myofibers of blAJ mice might arise from deficient activation of nicotinic acetylcholine receptors, which causes derepression of Cx expression in skeletal myofibers [40]. In agreement with this interpretation, presynaptic dysferlin deficiency has been shown to reduce the ACh release and promote muscle changes reminiscent of LGMD2B dysferlinopathy [41] and it has been shown that dysferlin plays a critical role in the trafficking of proteins [24]. Therefore, mutated dysferlin could lead to synaptic vesicles deficiency, creating a pseudo denervation condition. Opposite to this mechanism, boldine presents a weak inhibitory effect over acetylcholine esterase [42], which might increase the half-life of acetylcholine delaying the appearance of myofiber alteration promoted by a reduction in acetylcholine release. However, this putative effect might be canceled by the inhibitory effect of boldine on nicotinic receptors as it has been demonstrated in the mouse phrenic nerve-diaphragm [43]. In addition, the anti-oxidant effect of boldine [44] can be reinterpreted since several antioxidant compounds first block Cx HCs permeable to Ca^2+^ [45], which activates several intracellular metabolic pathways that generate reactive oxygen substances. Thus, it can be predicted that boldine’s first effect is to impede Ca^2+^ entrance and consequently prevents the generation of free radicals rather than acting directly as an anti-oxidant compound [45]. Therefore, and despite the pleiotropic effect of boldine at molecular components of the neuromuscular junction, the outcome of the boldine treatment might mainly reflect its action as a Cx HC inhibitor.

We have previously shown that de novo expression of Cx HCs in myofibers is directly associated with degeneration of these cells, favoring damage and loss of motor function, which was prevented in blAJ myofibers with Cx43 and Cx45 deficient expression [15]. Now, we demonstrated that symptomatic blAJ mice treated with boldine show complete recovery of motor function, indicating that inhibition of Cx HCs protects myofibers from undergoing damage, and restores the normal regeneration of myofibers. This was evidenced by the complete recovery of the number of mature myofibers with peripheral nuclei and normal CSA as well as a drastic reduction of serum CK activity. Interestingly, after about seven days of denervation, skeletal muscles present changes also found in dysferlinopathies, including the expression of the same Cxs that permeabilize the sarcolemma via Cx HCs. However, different to denervation, muscles bearing mutated dysferlin show intense muscle damage denoted by elevated serum CK activity. This suggests that muscle necrosis of dysferlinopathy is not solely due to the expression of Cx HCs. Nonetheless, the serum CK activity of blAJ mouse myofibers deficient in Cx43/Cx45 expression or blAJ mice treated with an inhibitor of Cx43 and Cx45 HCs was normal, implying that Cx HCs are involved in cell damage susceptibility. A radical difference between denervated and dysferlin mutated myofibers could be a nerve-activity dependent mechanism; since in dysferlin deficient individual, the attached nerve terminals can induce some muscle contraction whereas this cannot take place in denervated muscles.

Here, we confirmed that abolishing Cx43 and Cx45 HC activity with boldine, restores muscle function in a dysferlinopathy mouse model. This emphasizes each molecular step in which Cx HCs have been previously demonstrated to be involved, including the disappearance of dysferlin in myofibers. Specifically, the persistent increase in Ca^2+^ signal present in blAJ myofibers [15] was completely suppressed by treatment with boldine. Since in denervated myofibers a similar long-term increase in cytoplasmic Ca^2+^ signal activates proteases in a Cx43 and Cx45 HC-dependent manner [46], we propose that inhibition of these two Cx HCs in blAJ mice with boldine allow cells to recover their intracellular Ca^2+^ signaling to the normal level. The latter most likely reduces the Ca^2+^-dependent protease activity diminishing the degradation of dysferlin-like protein that becomes detectable by immunofluorescence and Western blot analysis. Notably, a dysferlin-like protein was also readily detected in blAJ myofibers deficient in Cx43/Cx45 expression [15].

The use of a Cx HC and gap junction channel blocker, such as Cbx, is not recommended because gap junction channels play important roles in normal functioning of several vital organs such as heart [47]. Therefore, chronic treatment with this compound could lead to heart arrhythmia and failure. Whereas, boldine blocks Cx HCs and does not affect gap junction channels [10,11], reducing its risk as a therapeutic agent to treat a chronic human disease. Since the etiology of dysferlinopathies is genetic, a putative therapeutic solution could be found using gene therapy (providing a normal copy of dysferlin) or gene repair (CRYSPR-Cas9). Alternatively, our findings strongly indicate that the unfolding of a cascade of events triggered by lack of functional dysferlin can be controlled without genetic approaches using a small organic molecule directed to a critical molecular target to overcome this invalidating and currently untreatable disease.

## 4. Materials and Methods

### 4.1. Reagents

N-benzyl-p-toluene sulphonamide (BTS), FURA 2-AM, carbenoxolone (Cbx), collagenase type I, and Evans blue (EB^4-^) were purchased from Merck (NJ, USA). Ethidium bromide (Etd^+^), DMEM/F12, and fetal bovine serum (FBS) were obtained from GIBCO/BRL (Grand Island, NY, USA). Fluoromont G plus DAPI was obtained from (Hatfield, PA, USA). Monoclonal anti-Cx43 antibody (1:250) was purchased from BD Biosciences (San Jose, CA, USA) and polyclonal anti-Cx45 (1:250) antibody was purchased from Invitrogen (Carlsbad, CA, USA), monoclonal anti-dysferlin (1:250) antibody was purchased from Cell Signaling (Danvers, MA, USA), polyclonal anti-PPARγ (1:300) antibody was obtained from Thermo Fisher (Waltham, MA, USA), anti-rabbit or anti-mouse IgG antibodies-conjugated to Cy2 (green) were obtained from Jackson immunoResearch Laboratories (West Grove, PA, USA). Boldine was prepared as described previously [8,10].

### 4.2. Animals

This proposal used males from two mouse strains: (1) wild type C57Bl/6 background; (2) blAJ mice (dysferlin-deficient animals). These animals bear a homozygous mutation in both alleles of the *DYSF* gene and present several muscle alterations characteristic of this type of muscular dystrophy [12]. When the animals were 8 months old, they were started on boldine treatment for 8 weeks. The blAJ animals were kindly donated by Jain Foundation.

### 4.3. Human Control and Dysferlin-Deficient Myoblast Cell Line

The cell line called AB1167, derived from a patient without muscular pathology was used as control and the cell line called 107, which is derived from human dysferlin-deficient myoblasts was used as in vitro model of dysferlinopathy [5]. Undetectable dysferlin expression in the 107 cell line was previously described [5]. Both cell lines were donated by Dr. Mouly (Paris, France). These cells were cultured in DMEM/F12 supplemented with 20% of fetal calf serum.

### 4.4. Isolation of Mouse Skeletal Myofibers

This protocol was obtained from [6]. Briefly, intact myofibers were dissociated from the flexor digitorum brevis (FDB) muscle (a fast muscle). The plantaris tendons and connective tissue were removed from anesthetized mice. Then, FDB muscles were dissected and incubated in culture medium (DMEM/F12 supplemented with 10% FBS) containing 0.2% collagenase type I, for 3 h at 37 °C, and transferred to test tube (Falcon) containing 3 mL of culture medium. FDB muscle was subsequently gently dissociated 15 times through a Pasteur pipette with a wide tip to disperse single myofibers. Dissociated myofibers were centrifuged at 1000 rpm for 15 s (model 8700 centrifuge; Kubota) and rinsed twice by sedimentation in PBS solution containing 10 μM N-benzyl-p-toluene sulfonamide (BTS), which reduced muscle damage (contractions inhibitor) during the isolation procedure. Finally, fibers were resuspended in 5 mL of Krebs HEPES buffer containing 10 μM BTS.

### 4.5. Boldine Treatment

The different mice strains used in this proposal were treated with boldine (100 mg/kg, daily) for 8 weeks. Powder boldine prepared as described [10] was administered to mice mixed with 3 mg of peanut butter in a separate cage (one mouse at the time). For cultures, Boldine final concentration was 50 µm.

### 4.6. Immunofluorescence Analysis

This protocol has been described in detail previously [6]. In brief, frozen cross-sections (10 μm thickness) were obtained from GC muscles and fixed with 4% formaldehyde for 10 min at room temperature. Cryosections were incubated for 3 h at room temperature in blocking and permeabilizing solution (50 mM NH_4_Cl, 0.025% Triton, 1% BSA on PBS solution 1×), incubated overnight with appropriate dilutions of primary antibody, rinsed four times with PBS, and then incubated by 1 h with secondary antibody conjugated to Cy2 or Cy3, and mounted in Fluoromount G plus DAPI. Positive immunoreactivity was detected with a Nikon Eclipse Ti microscope equipped with epifluorescence illumination, and images were obtained with a Clara camera (Andor).

Counterstaining with hematoxylin and eosin to tissue section was carried out as previously described [14].

### 4.7. Oil Red O Staining

This procedure was carried out as previously described [48] and was used to detect lipid accumulation in satellite cell line cultures or sections of skeletal muscles. Cross-cryosections (10 μm thickness) of fast-frozen tibialis anterior (TA) muscles were mounted on slides and fixed with baker solution (PFA plus 180 mM CaCl_2_) at 4 °C for 30 min. Then, samples were washed with water for 15 min to eliminate the excess of the fixator. Posteriorly, the slices were incubated with oil red O solution (5 g/L in 70% alcohol solution) at 37 °C during 15 min. Then, a second wash was performed. Finally, the muscle slices were incubated with a hematoxylin solution for 3 min and washed.

### 4.8. Western Blot Analysis

*GC* muscles were dissected and rinsed with ice-cold saline. Tendons were removed and muscles were cut in small pieces by using a razor blade. Then, the pieces were homogenized (Brinkmann homogenizer, Labequip Ltd.a, Ontario, Canada) and sonicated (Misonix inc., Farmingdale, NY, USA. The homogenates were centrifuged for 10 min at 13,000× *g* and pellets were discarded. The samples were then processed as previously described [49]. Nitrocellulose membranes were incubated overnight with appropriate dilutions of primary antibodies against dysferlin or PPARγ. Then, blots were rinsed with 1% PBS solution–Tween 20 and incubated for 40 min at room temperature (20–22 °C) with HRP-conjugated goat anti-rabbit IgGs (Santa Cruz Biotechnology, Dallas, TX, USA). After five rinses, immunoreactive proteins were detected using ECL reagents according to the manufacturer’s instructions (PerkinElmer, Waltham, MA, USA).

### 4.9. Evans Blue Uptake In Vivo

This protocol was described by [6]. Animals were injected i.p. 5 h before euthanasia with Evans blue (EB^4−^, 80 mg/kg) dissolved in a sterile saline solution. The in vivo EB^4−^ uptake by myofibers was inhibited by boldine (100 mg/kg) administered (i.p.) 20 min before the EB^4−^ injection. After this time, animals were euthanized and the GC muscles were dissected and fast-frozen in isopentane precooled in liquid nitrogen. EB^4−^ fluorescence intensity (λ excitation, 545 nm; λ emission, 595 nm) was quantified on cross-sections in the region of interest (center of myofibers) by using a conventional Nikon Eclipse Ti fluorescent microscope (EB^4−^ λ excitation, 545 nm; λ emission, 595 nm). The percentage of positive EB^4−^ myofibers was quantified.

### 4.10. Time-Lapse Recording of Etd^+^ Uptake

The Etd^+^ uptake was measured by time-lapse analysis as previously described [6,12]. In brief, freshly isolated myofibers were plated onto glass coverslips in a microscope chamber. Myofibers were incubated in recording medium containing 5 μM Etd^+^. The Etd^+^ fluorescence was analyzed in a region of interest located on different nuclei of myofibers. It was recorded by using water immersion Nikon eclipse Ti microscope, and the images were offline processed with ImageJ software (National Institutes of Health).

### 4.11. Intracellular Ca^2+^ Signal

The basal cytoplasmic Ca^2+^ amount, in isolated myofibers, was evaluated by the use of the ratiometric dye FURA 2-AM. The myofibers were incubated in Krebs-Ringer buffer (in mM: 145 NaCl, 5 KCl, 3 CaCl_2_, 1 MgCl_2_, 5.6 glucose, 10 HEPES-Na, pH7.4) by 55 min at room temperature. The Ca^2+^ amount was measured in a Nikon Eclipse Ti microscope equipped with epifluorescence illumination and images were obtained with a Clara camera (Andor) at two wavelengths (λ) 340 and 380 nm, calculating the ratio 340 vs. 380.

### 4.12. Cross-Sectional Area (CSA) Measurements

In cross-sections of GC muscles fixed with 4% (*wt*/*vol*) paraformaldehyde and stained with hematoxylin-eosin, the CSA of myofibers was evaluated by using off-line analyses by ImageJ software (National Institutes of Health).

### 4.13. Creatine Kinase (CK) Activity

Blood samples were obtained from different mice strains used. These samples (200–300 μL) were incubated (45–60 min) at 37 °C to wait for clot formation. Then, they were centrifuged (1000 rpm by 5 min) and the supernatant (serum) was transferred to a clean microtube. The enzymatic activity of serum creatine kinase was analyzed at 37 °C in a spectrophotometer (λ = 340 nm) following the manufacturer’s instructions (Valtek S.A., Santiago, Chile).

### 4.14. Physical Exercise Tests (Rotarod Test and Four Limbs Hanging Test)

Mice were subjected to running in the rotating bar of the IITC Rotarod apparatus (Life sciences) at a controlled velocity that was programmed to gradually increase the velocity. First, the animals were placed in a rotating bar without movement during 5 min to adapt to the equipment, and then animals were subjected to an increased g velocity protocol starting at 5 rotations per minute (rpm) until 20 rpm in a ramp of 80 s duration. Each week, on the same day, three trials were performed per animal with a rest period of 3 min between trials.

The four limbs hanging test was used to evaluate total skeletal muscle function and endurance over time. The set up was comprised of a metal grid positioned 40 cm above a flat surface covered with litter as fall caution (SOP DMD_M.2.1.005, G. Carlson). The time that mice remain hung was recorded until they fall. Both physical exercise measurements were blind tested.

## 5. Statistical Analyses

Results were presented as mean ± SEM. Two populations were compared by using the logarithm of ratio followed by the Student *t*-test. For multiple comparisons with a single control, a nonparametric one-way ANOVA followed by the Dunn test was used. Analyses were carried out by using GraphPad software. The sample size was 6 mice for each condition. *p* values < 0.05 were considered statistically significant.

## Supplementary Materials

The following are available online at https://www.mdpi.com/1422-0067/21/17/6025/s1, **Supplementary Figure S1.** Boldine blocks Cx45 but not Cx39 hemichannels. HeLa-Parental cells (HeLa-P) and HeLa cells transfected with Cx39 (HeLa Cx39) or connexin45 (HeLa Cx45) were used in time-lapse experiments to evaluate fluorescence intensity due to ethidium bromide (Etd^+^, 5 μM) uptake (in arbitrary units: AU). (**A**–**C**) The basal values were evaluated for 10 min. Then, the opening of connexin hemichannels was induced by exposure to the divalent cation-free solution (DCFS) and after 10 min recording, cells were treated with 50 µM boldine. (**D**) Graphs show the Etd^+^ uptake rates [Arbitrary Units (AU)/min] under conditions described for (**A**–**C**). Each plotted value represents the mean ± SEM. (between 30 and 50 cells were recorded in each experiment; *n* = 6 cell cultures). *** *p* < 0.0005; no significance (n.s.) *p* > 0.05; **Supplementary Figure S2.** Myofibers of skeletal muscles from dysferlin deficient mice present functional connexin hemichannels in vivo. Five hours after Evans blue (EB^4-^) injection (80 mg/kg), GC muscles were dissected from 8 weeks old control, dysferlin-deficient (blAJ) and blAJ mice treated with boldine (8 weeks) and processed for fluorescence evaluation. The inset shows a graph with the quantification of intracellular EB^4-^ staining (red signal inside the myofibers). *N* = 6 animals for each condition. *** *p*< 0.001 compared to all conditions studied. Scale bar = 50 μm; **Supplementary Figure S3.** Boldine normalizes the sarcoplasm permeability and cytoplasmic Ca^2+^ signal of ex vivo myofibers from blAJ mice. Myofibers were isolated from FDB muscles of control (wt, white bar), control treated with boldine (Control + boldine; 8 weeks treatment with a daily dose of 100 mg/kg boldine, grey bar), dysferlin-deficient (blAJ; black bar) and blAJ mice treated with boldine (A/J + boldine; green bar). (**A**) The connexin hemichannel activity was evaluated using the ethidium (Etd^+^) uptake assay. The Etd^+^ uptake rate corresponds to the slope of the Etd^+^ fluorescence intensity evaluated in time-lapse experiments. (**B**) The same animal groups as in A were additionally treated or not with 50 μM carbenoxolone (Cbx). In FDB myofibers loaded with FURA-2, the cytoplasmic Ca^2+^ signal was evaluated in freshly isolated myofibers. (**C**) Graph showing Ca^2+^ signal evaluated in myofibres from control (white bar), control treated with boldine (100 mg/kg, black bar), blAJ (grey bar), and blAJ mice treated during 8 weeks with boldine (100 mg/kg, green bar) mice. Each value in both graphs represents the mean ± SEM. *N* = 6 animals for each condition with 20 fibers per animal. ** *p* < 0.01 and *** *p* < 0.001 respect to all studied conditions; **Supplementary Figure S4.** Boldine reverts the nuclei distribution and the cross-sectional area of myofibers in dysferlin deficient mice. GC muscles were dissected from control (white bar), control treated with boldine (inset, 100 mg/kg daily during 8 weeks, grey bar), dysferlin-deficient (blAJ, black bar) and blAJ mice treated with boldine (100 mg/kg daily during 8 weeks, green bar). (**A**) Cross-sections of muscles were counterstained and, in each field, peripheral and internal nuclei are denoted with black and light green arrows, respectively. (**B**) and (**C**) graphs showing the number of myofibers with internal nuclei and CSA, respectively, under each condition. *N* = 6 animals for each condition. Each value represents the mean ± SEM, *n* = 6; * *p* < 0.05 and ** *p* < 0.01.

## Abbreviations

PBS	Phosphate-buffered saline
Cx	Connexin
Cx43 HC	Connexin43 hemichannel
Cx45 HC	Connexin45 hemichannel.
Cx39 HCs	Connexin39 hemichannels.
EB^4-^	Evans blue
Etd^+^	Ethidium bromide
CSA	Cross-sectional area
CK	Creatine Kinase
Cbx	Carbenoxolone
GC	Gastrocnemius
MHC	Myosin Heavy Chain
SEM	Standard error of the mean

## References

[1] Bansal D., Miyake K., Vogel S.S., Groh S., Chen C.C., Williamson R., McNeil P.L., Campbell K.P. (2003). Defective membrane repair in dysferlin-deficient muscular dystrophy. Nature.

[2] Lostal W., Bartoli M., Roudaut C., Bourg N., Krahn M., Pryadkina M., Borel P., Suel L., Roche J.A., Stockholm D. (2012). Lack of correlation between outcomes of membrane repair assay and correction of dystrophic changes in experimental therapeutic strategy in dysferlinopathy. PLoS ONE.

[3] Gómez-Andrés D., Díaz J., Munell F., Sánchez-Montáñez Á., Pulido-Valdeolivas I., Suazo L., Garrido C., Quijano-Roy S., Bevilacqua J.A. (2019). Disease duration and disability in dysferlinopathy can be described by muscle imaging using heatmaps and random forests. Muscle Nerve.

[4] Haynes V.R., Keenan S.N., Bayliss J., Lloyd E.M., Meikle P.J., Grounds M.D., Watt M.J. (2019). Dysferlin deficiency alters lipid metabolism and remodels the skeletal muscle lipidome in mice. J. Lipid Res..

[5] Cea L.A., Bevilacqua J.A., Arriagada C., Cárdenas A.M., Bigot A., Mouly V., Sáez J.C., Caviedes P. (2016). The absence of dysferlin induces the expression of functional connexin-based hemichannels in human myotubes. BMC Cell Biol..

[6] Cea L.A., Cisterna B.A., Puebla C., Frank M., Figueroa X.F., Cardozo C., Willecke K., Latorre R., Sáez J.C. (2013). De novo expression of connexin hemichannels in denervated fast skeletal muscles leads to atrophy. Proc. Natl. Acad. Sci. USA.

[7] Cea L.A., Balboa E., Puebla C., Vargas A.A., Cisterna B.A., Escamilla R., Regueira T., Sáez J.C. (2016). Dexamethasone-induced muscular atrophy is mediated by functional expression of connexin-based hemichannels. Biochim. Biophys. Acta.

[8] Cea L.A., Balboa E., Vargas A.A., Puebla C., Brañes M.C., Escamilla R., Regueira T., Sáez J.C. (2019). De novo expression of functional connexins 43 and 45 hemichannels increases sarcolemmal permeability of skeletal myofibers during endotoxemia. Biochim. Biophys. Acta Mol. Basis Dis..

[9] D’hondt C., Ponsaerts R., De Smedt H., Bultynck G., Himpens B. (2009). Pannexins, distant relatives of the connexin family with specific cellular functions?. Bioessays.

[10] Hernández-Salinas R., Vielma A.Z., Arismendi M.N., Boric M.P., Sáez J.C., Velarde V. (2013). Boldine prevents renal alterations in diabetic rats. J. Diabetes Res..

[11] Yi C., Ezan P., Fernández P., Schmitt J., Sáez J.C., Giaume C., Koulakoff A. (2017). Inhibition of glial hemichannels by boldine treatment reduces neuronal suffering in a murine model of Alzheimer’s disease. Glia.

[12] Lostal W., Bartoli M., Bourg N., Roudaut C., Bentaib A., Miyake K., Guerchet N., Fougerousse F., McNeil P., Richard I. (2010). Efficient recovery of dysferlin deficiency by dual adeno-associated vector-mediated gene transfer. Hum. Mol. Genet..

[13] Grounds M.D., Terrill J.R., Radley-Crabb H.G., Robertson T., Papadimitriou J., Spuler S., Shavlakadze T. (2014). Lipid accumulation in dysferlin-deficient muscles. Am. J. Pathol..

[14] Agarwal A.K., Tunison K., Mitsche M.A., McDonald J.G., Garg A. (2019). Insights into lipid accumulation in skeletal muscle in dysferlin-deficient mice. J. Lipid Res..

[15] Fernández G., Arias G.B., Bevilacqua J.A., Castillo M., Caviedes P., Sáez J.C., Cea L.A. (2020). Myofibers deficient in connexins 43 and 45 expression protect mice from skeletal muscle and systemic dysfunction promoted by a dysferlin mutation. Biochem Biophys Acta Mol. Basis Dis.

[16] Proulx A., Merrifield P.A., Naus C.C. (1997). Blocking gap junctional intercellular communication in myoblasts inhibits myogenin and MRF4 expression. Dev. Genet..

[17] Burr A.R., Molkentin J.D. (2015). Genetic evidence in the mouse solidifies the calcium hypothesis of myofiber death in muscular dystrophy. Cell Death Differ..

[18] Farini A., Sitzia C., Navarro C., D’Antona G., Belicchi M., Parolini D., Del Fraro G., Razini P., Bottinelli R., Meregalli M. (2012). Absence of T and B lymphocytes modulates dystrophic features in dysferlin deficient animal model. Exp. Cell Res..

[19] Galassi G., Rowland L.P., Hays A.P., Hopkins L.C., Di Mauro S. (1987). High serum levels of creatine kinase: Asymptomatic prelude to distal myopathy. Muscle Nerve.

[20] Haddix S.G., Lee Y.I., Kornegay J.N., Thompson W.J. (2018). Cycles of myofiber degeneration and regeneration lead to remodeling of the neuromuscular junction in two mammalian models of Duchenne muscular dystrophy. PLoS ONE.

[21] Ho M., Post C.M., Donahue L.R., Lidov H.G.W., Bronson R.T., Goolsby H., Watkins S.C., Cox G.A., Robert Brown R.J. (2004). Disruption of muscle membrane and phenotype divergence in two novel mouse models of dysferlin deficiency. Hum. Mol. Genet..

[22] Barry M.E., Pinto-González D., Orson F.M., McKenzie G.J., Petry G.R., Barry M.A. (1999). Role of endogenous endonucleases and tissue site in transfection and CpG-mediated immune activation after naked DNA injection. Hum. Gene.

[23] Mukherjee R., Das A., Chakrabarti S., Chakrabarti O. (2017). Calcium dependent regulation of protein ubiquitination—Interplay between E3 ligases and calcium binding proteins. Biochim. Biophys. Acta Mol. Cell Res..

[24] Leung C., Utokaparch S., Sharma A., Yu C., Abraham T., Borchers C., Bernatchez P. (2011). Proteomic identification of dysferlin-interacting protein complexes in human vascular endothelium. Biochem. Biophys. Res. Commun..

[25] Ryall J.G., Dell’Orso S., Derfoul A., Juan A., Zare H., Feng X., Clermont D., Koulnis M., Gutierrez-Cruz G., Fulco M. (2015). The NAD(+)-dependent SIRT1 deacetylase translates a metabolic switch into regulatory epigenetics in skeletal muscle stem cells. Cell Stem Cell.

[26] Ramachandran S., Xie L.-H., John S.A., Subramaniam S., Lal R. (2007). A Novel Role for Connexin Hemichannel in Oxidative Stress and Smoking-Induced Cell Injury. PLoS ONE.

[27] Aguiari P., Leo S., Zavan B., Vindigni V., Rimessi A., Bianchi K., Franzin C., Cortivo R., Rossato M., Vettor R. (2008). High glucose induces adipogenic differentiation of muscle-derived stem cells. Proc. Natl. Acad. Sci. USA.

[28] Chi J., Li L., Liu M., Tan J., Tang C., Pan Q., Wang D., Zhang Z. (2012). Pathogenic Connexin-31 Forms Constitutively Active Hemichannels to Promote Necrotic Cell Death. PLoS ONE.

[29] Wang C., Liu W., Nie Y., Qaher M., Horton H.E., Yue F., Asakura A., Kuang S. (2017). Loss of MyoD promotes fate transdifferentiation of myoblasts into Bbrown adipocytes. EBioMedicine.

[30] Chiu Y.-H., Hornsey M.A., Klinge L., Jørgensen L.H., Laval S.H., Charlton R., Barresi R., Straub V., Lochmüller H., Bushby K. (2009). Attenuated muscle regeneration is a key factor in dysferlin-deficient muscular dystrophy. Hum. Mol. Genet..

[31] Galvin J.E., Palamand D., Strider J., Milone M., Pestronk A. (2006). The muscle protein dysferlin accumulates in the Alzheimer brain. Acta Neuropathol..

[32] De Vuyst E., Decrock E., Cabooter L., Dubyak G.R., Naus C.C., Evans W.H., Leybaert L. (2006). Intracellular calcium changes trigger connexin 32 hemichannel opening. Embo J..

[33] Retamal M.A., Schalper K.A., Shoji K.F., Bennett M.V.L., Sáez J.C. (2007). Opening of connexin 43 hemichannels is increased by lowering intracellular redox potential. Proc. Natl. Acad. Sci. USA.

[34] Figueroa X.F., Lillo M.A., Gaete P.S., Riquelme M.A., Sáez J.C. (2013). Diffusion of nitric oxide across cell membranes of the vascular wall requires specific connexin-based channels. Neuropharmacology.

[35] Rawat R., Cohen T.V., Ampong B., Francia D., Henriques-Pons A., Hoffman E.P., Nagaraju K. (2010). Inflammasome up-regulation and activation in dysferlin-deficient skeletal muscle. Am. J. Pathol..

[36] Retamal M.A., Froger N., Palacios-Prado N., Ezan P., Sáez P.J., Sáez J.C., Giaume C. (2007). Cx43 hemichannels and gap junction channels in astrocytes are regulated oppositely by proinflammatory cytokines Released from Activated Microglia. J. Neurosci..

[37] Morozzi G., Beccafico S., Bianchi R., Riuzzi F., Bellezza I., Giambanco I., Arcuri C., Minelli A., Donato R. (2017). Oxidative stress-induced S100B accumulation converts myoblasts into brown adipocytes via an NF-κB/YY1/miR-133 axis and NF-κB/YY1/BMP-7 axis. Cell Death Differ..

[38] Gu W., Schneider J.W., Condorelli G., Kaushal S., Mahdavi V., Nadal-Ginard B. (1993). Interaction of myogenic factors and the retinoblastoma protein mediates muscle cell commitment and differentiation. Cell.

[39] Shao D., Lazar M.A. (1997). Peroxisome proliferator activated receptor gamma, CCAAT/enhancer-binding protein alpha, and cell cycle status regulate the commitment to adipocyte differentiation. J. Biol. Chem..

[40] Cisterna B.A., Vargas A.A., Puebla C., Fernández P., Escamilla R., Lagos C.F., Matus M.F., Vilos C., Cea L.A., Barnafi E. (2020). Active acetylcholine receptors prevent the atrophy of skeletal muscles and favor reinnervation. Nat. Commun..

[41] Krajacic P., Pistilli E.E., Tanis J.E., Khurana T.S., Lamitina S.T. (2013). FER-1/Dysferlin promotes cholinergic signaling at the neuromuscular junction in C. elegans and mice. Biol. Open.

[42] Kostelnik A., Pohanka M. (2018). Inhibition of acetylcholinesterase and butyrylcholinesterase by a plant secondary metabolite boldine. Biomed. Res. Int..

[43] Kang J.J., Cheng Y.-W., Fu W.-M. (1998). Studies on neuromuscular blockade by boldine in the mouse phrenic-diafragm. Jpn. J. Pharm..

[44] O’Brien P., Carrasco-Pzo C., Speisky H.C. (2006). Boldine and its antioxidant or health-promoting properties. Chem. Biol. Interact..

[45] Balboa E., Saavedra F., Cea L.A., Ramírez V., Escamilla R., Vargas A.A., Regueira T., Sáez J.C. (2020). Vitamin E Blocks Connexin Hemichannels and Prevents Deleterious Effects of Glucocorticoid Treatment on Skeletal Muscles. Int. J. Mol. Sci..

[46] Cisterna B.A., Vargas A.A., Puebla C., Sáez J.C. (2016). Connexin hemichannels explain the ionic imbalance and lead to atrophy in denervated skeletal muscles. Biochim. Biophys. Acta.

[47] Kurtenbach S., Kurtenbach S., Zoidl G. (2014). Gap junction modulation and its implications for heart function. Front. Physiol..

[48] Koopman R., Schaart G., Hesselink M.K. (2001). Optimisation of Oil Red O Staining Permits Combination With Immunofluorescence and Automated Quantification of Lipids. Histochem. Cell Biol..

[49] Riquelme M.A., Cea L.A., Vega J.L., Boric M.P., Monyer H., Bennett M.V., Frank M., Willecke K., Sáez J.C. (2013). The ATP required for potentiation of skeletal muscle contraction is released via pannexin hemichannels. Neuropharmacology.

