# Supplementary figures and images for: Blockade of Hemichannels Normalizes the Differentiation Fate of Myoblasts and Features of Skeletal Muscles from Dysferlin-Deficient Mice
Source: Int J Mol Sci. 2020 Aug 21;21(17):6025. doi: 10.3390/ijms21176025 (PMC7503700; doi:10.3390/ijms21176025)

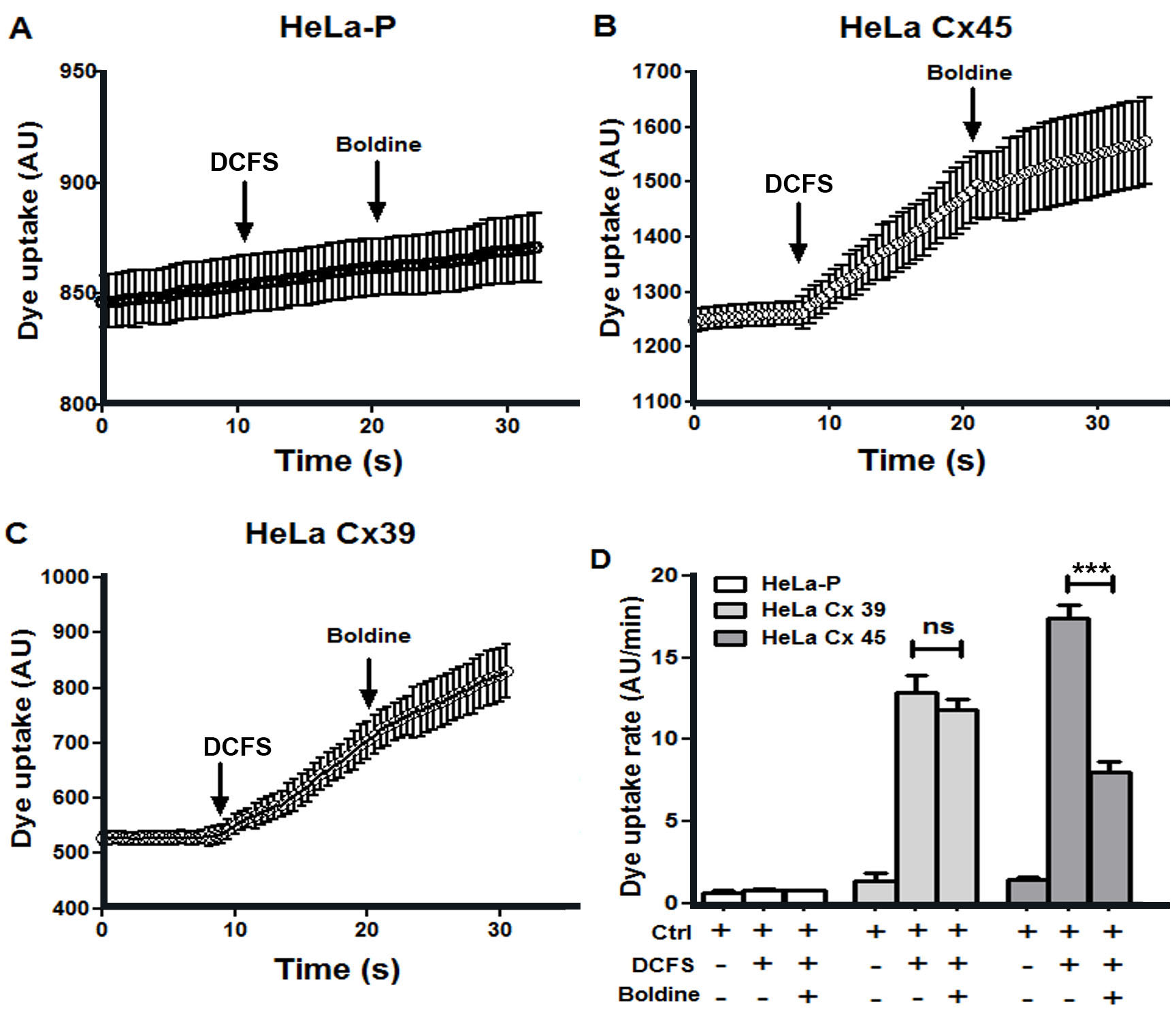

Supplement: Supplementary file 1 [file ijms-21-06025-s001.zip › Supplementary figures/Suppl Fig 1.jpg]

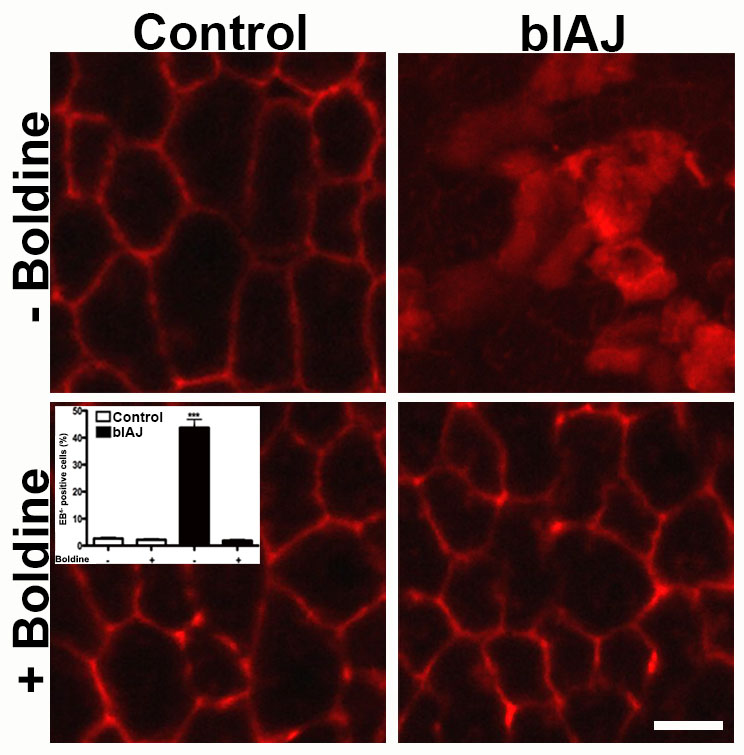

Supplement: Supplementary file 1 [file ijms-21-06025-s001.zip › Supplementary figures/Suppl Fig 2.jpg]

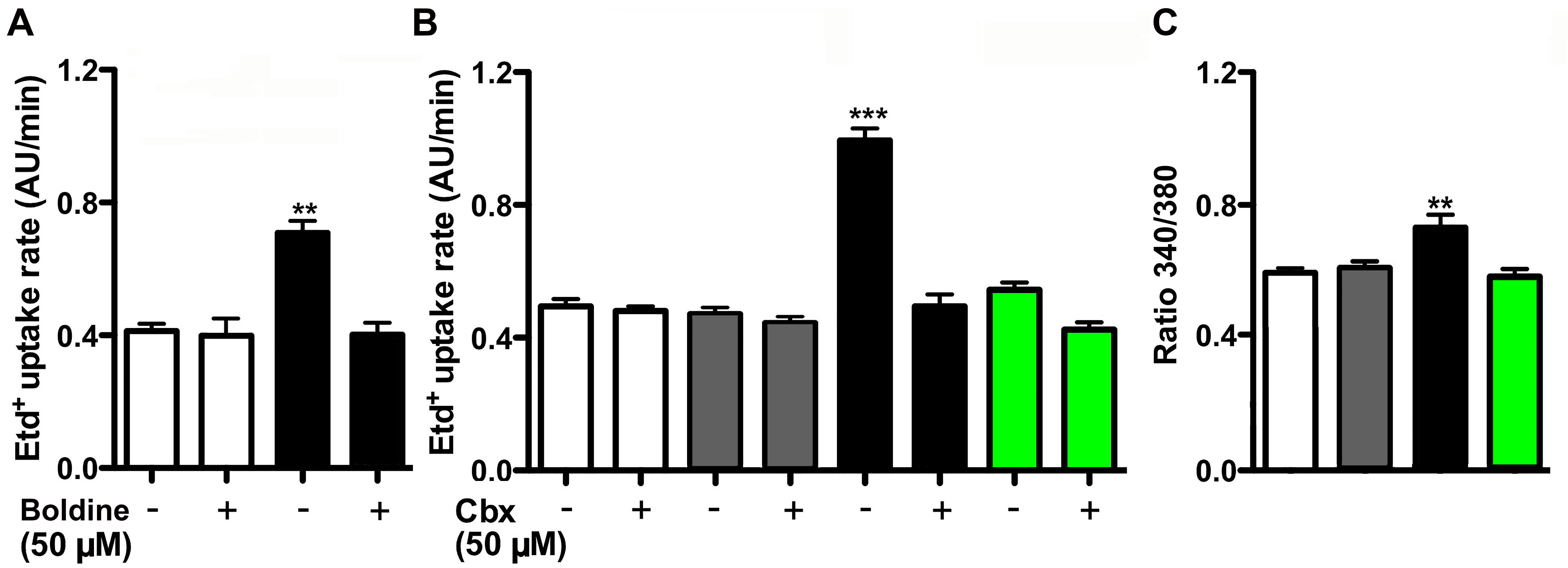

Supplement: Supplementary file 1 [file ijms-21-06025-s001.zip › Supplementary figures/Suppl Fig 3.jpg]

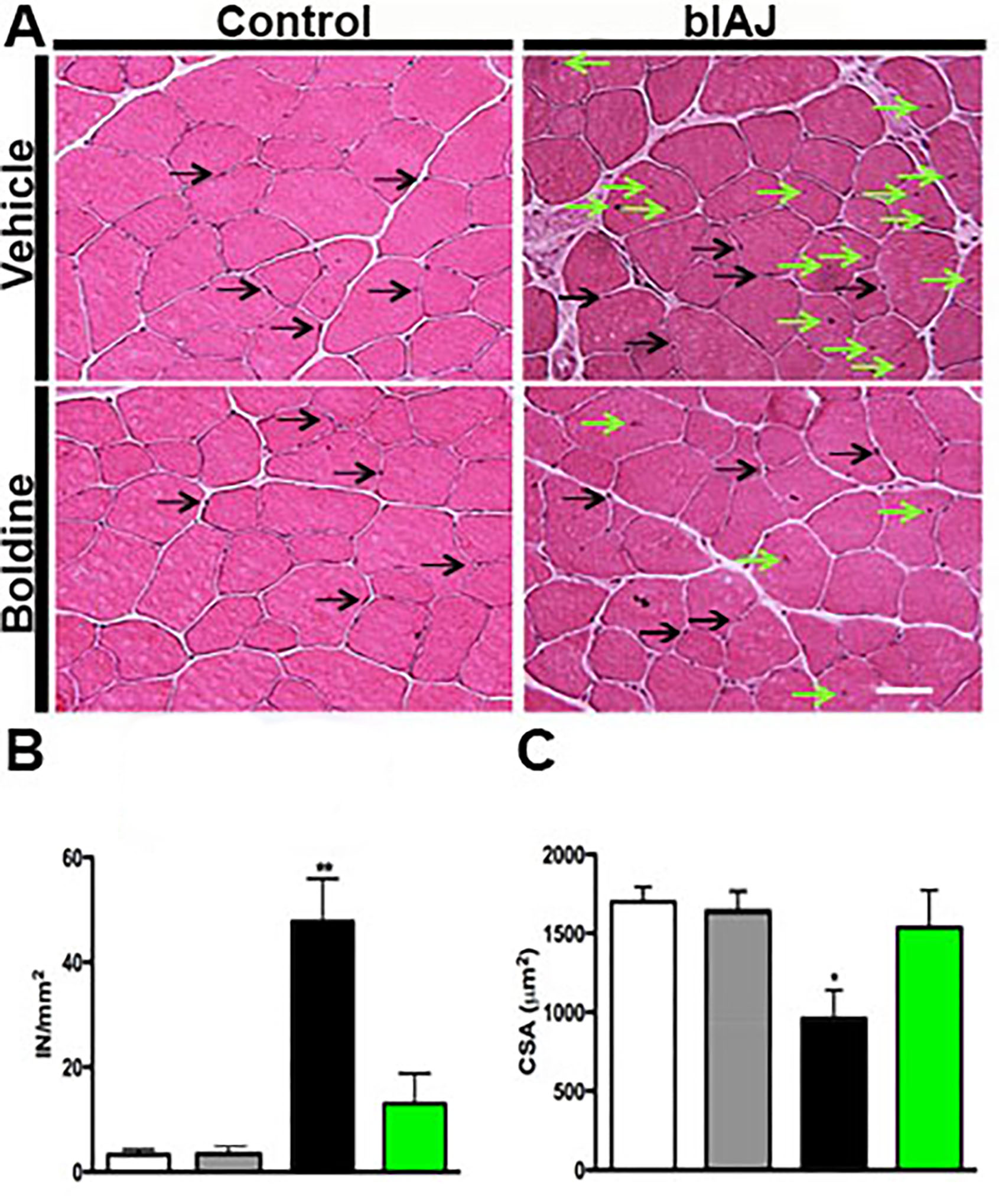

Supplement: Supplementary file 1 [file ijms-21-06025-s001.zip › Supplementary figures/Suppl Fig 4.jpg]

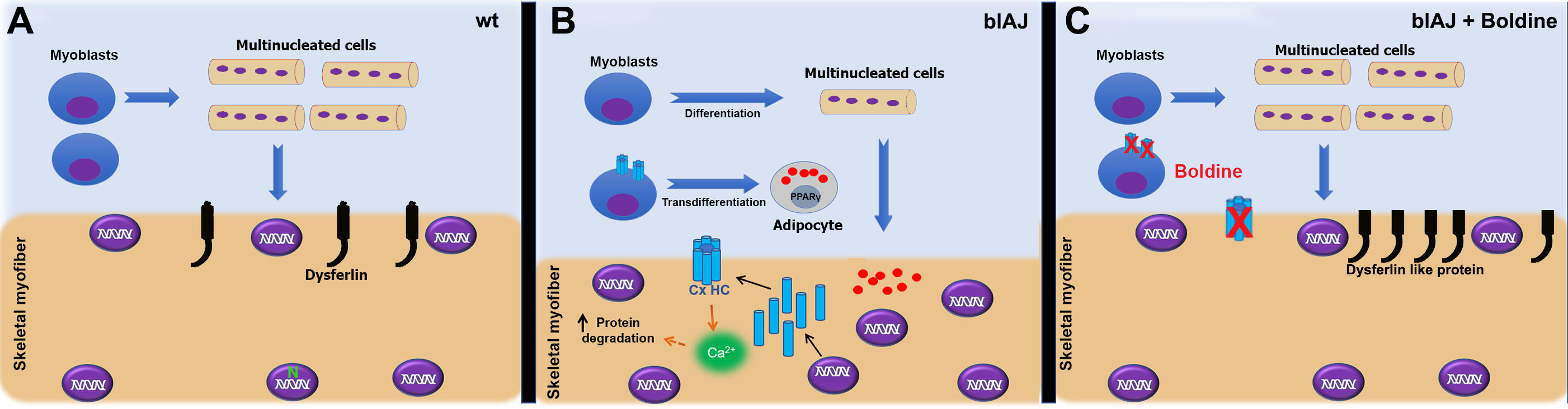

Supplement: Supplementary file 1 [file ijms-21-06025-s001.zip › Supplementary figures/Suppl Fig 5.jpg]
